# Beneficial effects of ascorbic acid to treat lung fibrosis induced by paraquat

**DOI:** 10.1371/journal.pone.0205535

**Published:** 2018-11-05

**Authors:** Marcia Rodrigues da Silva, Adriana Schapochnik, Mayara Peres Leal, Janete Esteves, Cristina Bichels Hebeda, Silvana Sandri, Christiane Pavani, Anna Carolina Ratto Tempestini Horliana, Sandra H. P. Farsky, Adriana Lino-dos-Santos-Franco

**Affiliations:** 1 Post Graduate Program in Biophotonics Applied to Health Sciences, University Nove de Julho (UNINOVE), São Paulo, Brazil; 2 Department of Clinical and Toxicological Analyses, Faculty of Pharmaceutical Sciences, University of São Paulo, São Paulo, Brazil, Brazil; Centre National de la Recherche Scientifique, FRANCE

## Abstract

Paraquat (PQ) is one of the most widely employed herbicides that is used worldwide and it causes severe toxic effects in humans and animals. A PQ exposition can lead to pulmonary fibrosis (PF) and the mechanisms seem to be linked to oxidative stress, although other pathways have been suggested. Antioxidants can be useful as a therapy, although interventions with this kind of system are still controversial. Hence, this study has investigated the role of ascorbic acid (vitamin C) post-treatment on PQ-induced PF in male C57/BL6 mice. Pulmonary fibrosis was induced by a single PQ injection (10mg/kg; i.p.). The control group received a PQ vehicle. Seven days after the PQ or vehicle injections, the mice received vitamin C (150 mg/kg, ip, once a day) or the vehicle, over the following 7 days. Twenty-four hours after the last dose of vitamin C or the vehicle, the mice were euthanized and their bronchoalveolar lavage fluid (BALF) and their lungs were collected. The data obtained showed that vitamin C reduced the cellular recruitment, the secretion of IL-17 –a cytokine involved in neutrophils migration, TGF-β–a pro-fibrotic mediator and the collagen deposition. Moreover, vitamin C elevated the superoxide dismutase (SOD) and catalase levels, both antioxidant enzymes, but it did not alter the tracheal contractile response that was evoked by methacholine. Therefore, the researchers have highlighted the mechanisms of vitamin C as being non-invasive and have suggested it as a promising tool to treat lung fibrosis when it is induced by a PQ intoxication.

## Introduction

Paraquat (N,N-dimethyl-4,4'-bipyridinium dichloride; PQ) is one of the most extensively used herbicides in the world, even though it causes severe toxic effects in humans and animals [1]. The toxic effects of PQ affect many tissues, such as the brain, the kidneys and the liver. Nevertheless, the toxicity that is caused in the lungs is more pronounced in accidental or intentional acute or long term environmental/occupational intoxications. A PQ exposure induces an acute lung injury (ALI) that is characterized by severe hypoxia, edema and dysfunction at the respiratory level. Subsequently, an ALI can progress to pulmonary fibrosis, causing a permanent loss of lung tissue [2,3,4]. The mortality rate of PQ poisoning is as high as 60%–80%, due to the lack of an effective therapy [2,3,5].

It has been proposed that the toxic potential of PQ results from its ability to produce reactive oxygen species and reactive nitrogen species, through the redox cycling processes, resulting in mitochondrial oxidative stress and potential cell death [6,7,8,9]. These mechanisms have been proposed as being pivotal to a PQ-induced lung injury. This has been extended, as high levels of mitochondria DNA fragments have been found in the bronchoalveolar lavage fluids of PQ exposed mice and these fragments have triggered the innate immune system that is involved in lung inflammation. These mechanisms have been reinforced by the reduced lung lesions in animals that were treated with DNase I, a nuclease that is able to cut and fragment the DNA molecules [10]. Moreover, it has recently been shown that PQ-induced lung fibrosis was dependent on other intracellular pathways that are involved in the phenotypic switching of the alveolar epithelial cells to fibroblasts [11,12,13,14]. Hence, the mechanisms of PQ-induced lung lesions are complex and they have not yet been totally identified.

PF is a chronic and progressive lung disease that is characterized by progressive lesions of the lung parenchyma, the inflammatory infiltrate and the interstitial fibrosis [15]. Clinical studies have suggested that oxidative stress plays an important role in PF, as elevated levels of the reactive oxygen species are accompanied by reduced anti-oxidant protein levels [16,17,18]. Furthermore, the redox status of the lung cells modulates the extracellular matrix (ECM) expression, which involves the migration of cells and the remodeling of the tissues [19]. Indeed, oxidative stress-mechanisms trigger the expression levels of the pro-fibrotic factors [20,21]. The rescue modes of a lung function are associated with reduced levels of oxidative stress and antioxidants, therefore, can be employed as a therapy to restore the lung homeostasis. Although, the employments of antioxidants are remarkable tools, in order to reduce the lung injuries in experimental animals [22,23], the clinical use of them to treat PF has not been readily accepted [24].

Vitamin C (ascorbic acid) is a potent antioxidant that contributes to the immune defense system, supporting several cellular functions of the innate and the adaptive immune system. It forms an epithelial barrier against the pathogens and it promotes the elimination of oxidants in the skin, thus, protecting against oxidative environmental stress. Vitamin C rapidly donates electrons, which impairs the damages of the biomolecules of the oxidants [25]. It is also a cofactor for several enzymes, such as the monooxygenase and the dioxygenase enzymes. This is in addition to the lysyl and prolyl hydroxylases that are essential for the stabilization of collagen, together with other hydroxylases that are involved in carnitine biosynthesis, which are pivotal for the transportation of fatty acids into the mitochondria from the generation of metabolic energy [26,27].

In this study, the researchers have aimed at investigating the role of oxidative stress in pulmonary fibrosis that was caused as the result of an acute PQ intoxication, by studying the effects of vitamin C on several parameters of lung lesions. The data has clearly shown that oxidative stress was the main mechanism of lung fibrosis that was caused by an acute intoxication of PQ in the studied mice and that vitamin C may be a remarkable tool for treating this type of an intoxication.

## Materials and methods

### Animals

Male mice (C57BL/6) were obtained from the University Nove de Julho, and maintained in a light and temperature-controlled room (12/12-hour light-dark cycle, 21 ± 2°C), with free access to food and water. The experiments were approved by the Animal Care Committee University Nove de Julho (CoEP-UNINOVE, AN0017/2016).

### Pulmonary fibrosis induced by paraquat (PQ)

Pulmonary fibrosis was induced by single PQ administration (10 mg/kg, ip) and the analyses were performed 15 days after the PQ injection. Control animals received sterile saline. The period of 15 days was chosen considering that this is the period required to the establishment of lung fibrosis [28,29].

### Vitamin C treatment

Seven days after PQ injection, mice were treated with vitamin C (150mg/kg, ip, once a day) or sterile saline during 7 days according to Ibrahim et al. [30]. Twenty-four hours after the last injection, mice were euthanized by sectioning the abdominal aorta under deep anesthesia (ketamine and xylazine; 100mg/kg and 20mg/kg, respectively), and samples were collected for subsequent analyses.

### Groups of study

Animals were randomised as fibrosis (PQ injection and vitamin C vehicle), named as F; vitamin C treated, named as F+Vit C and Basal group (non-manipulated mice), named as B. The control group (PQ vehicle) was carried out in all experimental procedures and, data obtained was similar to the Basal group. For this reason, data of control group was abrogated from results. In this study 3 sets of experiments were carried out, using 2 animals in each group, totalizing 6 animals per group. Data were grouped and presented as mean of them. Please see Table 1.

**Table 1:** Groups of study.

Treatments	Groups
Non-manipulated mice	B
Fibrotic mice	F
Fibrotic mice treated with Vitamin C	F+VitC

### Cells quantification into the bronchoalveolar lavage fluid (BALF)

In order to determine the number of cells recruited into the alveolar space, the tracheae of mice were cannulated, and the alveolar space were flushed twice with Phosphate buffered saline (PBS, 1,5 ml total volume). The collected BALF was centrifuged (1500 rpm for 15 min at 20 C), and the resulting cell pellet was resuspended in 1 ml of PBS. The cell suspension was stained with crystal violet, and the total cell number was determined microscopically using a Neubauer chamber. The phenotypic of leukocytes were carried out via cytospin preparations and staining with Instant-Prov.

We also analyzed the phenotypic leukocytes by flow cytometry. In summary, BALF cells were washed in PBS with bovine serum albumin 1% (PBS/BSA) and further incubated with monoclonal antibodies anti-CD3 FITC, anti-CD11b and anti-Ly6G PerCP (Becton Dickinson—BD, East Rutherford, NJ, USA) for 20 minutes, at room temperature. Samples were acquired in BD Accuri C6 flow cytometer and 10.000 events were considered for analyses using CSampler software (Becton Dickinson—BD, East Rutherford, NJ, USA).

### Quantification of inflammatory and pro-fibrotic mediators as well as antioxidant enzymes in the lung homogenates

Levels of inflammatory mediators (IL-6, TNF-α, IL-17), pro-fibrotic (MMP-9, TGF-β), and antioxidant enzymes (superoxide dismutase (SOD) and catalase (CAT) were determined in the lung homogenates by ELISA, according manufacturer’s instructions (Biolegend, Mybiosource, San Diego, USA, and Cayman, Michigan, USA). The results were expressed as pg of mediator or enzyme produced per mg of tissue.

### Evaluation of collagen production in the lung

Lung fragments were fixed in paraformaldehyde (4%) for 24 h, dehydrate in alcohol and diaphanizated with xylene. In sequence, the fragments were embedded in paraffin, deparaffinized, and sectioned at 5 μm (microtome HYRAX M60, Zeiss, GR). Then, the tissues were stained with Picrossirius for collagen analysis and the quantification was performed by Image-Pro plus 7 (Media Cybernetics, Inc, USA).

### Maximum contractile response of tracheal tissue

In order to investigate the effects of Vitamin C treatment on smooth muscle contraction, isometric force development was quantified in tracheal rings mounted in a 15-ml organ bath by means of two steel hooks. Force contraction was recorded using a force displacement transducer and a chart recorder (Powerlab, Labchart, AD Instruments). Tracheal rings were suspended in organ bath filled with continuously aerated (95% O2 and 5% CO2) Krebs-Hanseleit (KH) solution at 37 C. After 40-min, the tracheal tension was adjusted to 0.5 g e was added methacholine (MCh, 10^-3^M) in order to obtain maximum contractile response.

### Statistical analyses

Data were expressed as the means ± SEM, and comparisons among the experimental groups were analyzed by one-way ANOVA followed by the Student´s Newman-Keuls test for multiple comparisons using the GraphPad software V.5. P-values less than 0.05 were considered statistically significant.

## Results

### Vitamin C treatment reduced the number of neutrophils, macrophages and lymphocytes into the BALF of fibrotic mice

**Fig 1:**
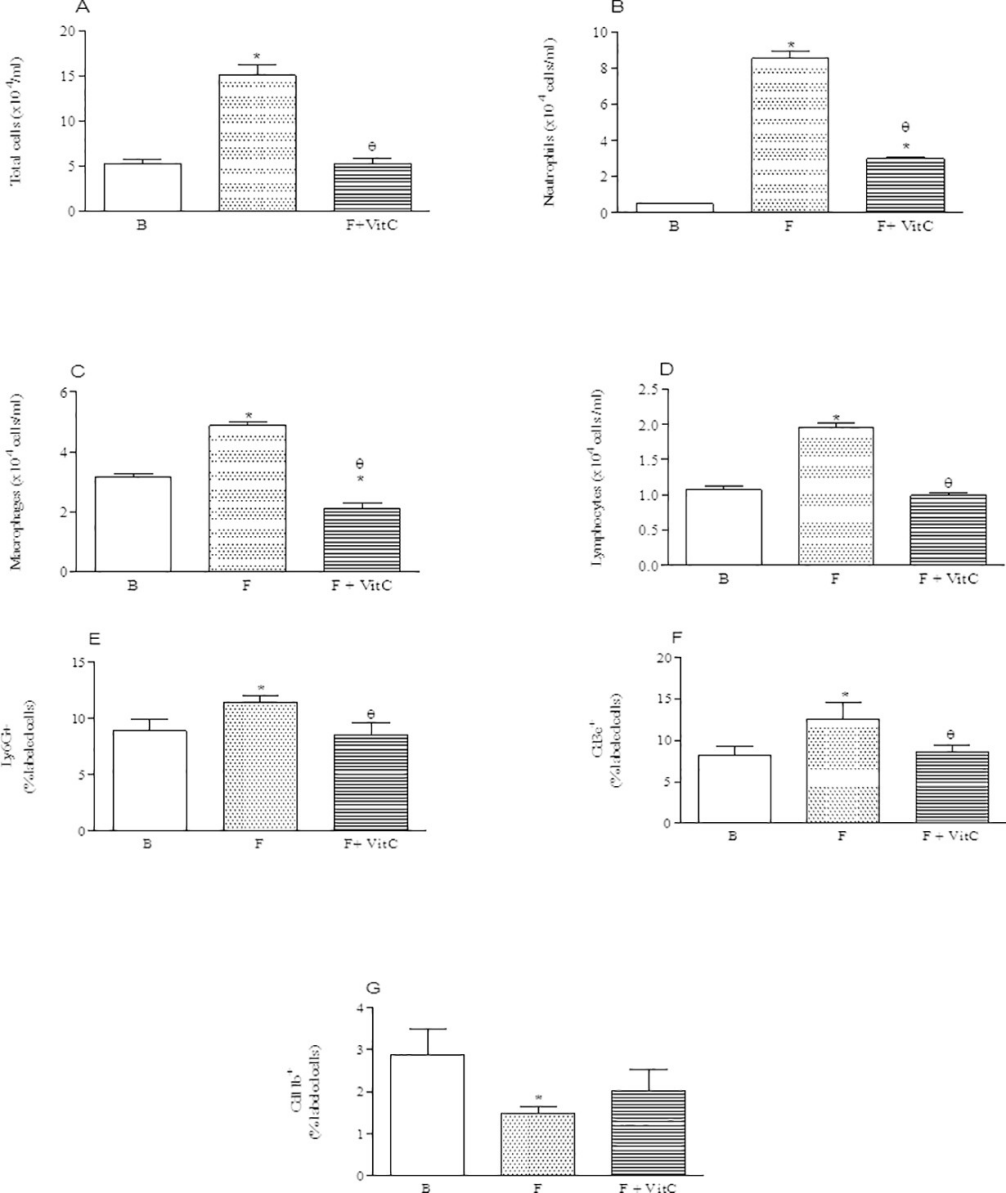
Vitamin C treatment reduces cell influx in the BALF of PQ-induced fibrosis. Groups of mice were submitted to PQ or vehicle injection and after 7 days the animals were treated with vitamin C or vehicle daily until day 14th. Twenty-four hours after the last vitamin C injection the total number and differential cells recovered in the bronchoalveolar lavage was determined. Data represent the mean ± SEM of 6 animals. *P<0.05 in relation to B group; ^θ^P <0.05 in relation to F group.

Cell recruitment is an important hallmark for the development of pulmonary fibrosis. Our data confirmed the elevated number of total cells in fibrotic animals PQ-induced when compared to basal group. Treatment with vitamin C abrogated the increased leukocytes number in the BALF observed in the fibrotic group (Fig 1, Panel A).

The phenotypic analysis of leukocytes by cytospin showed increased number of neutrophils in fibrotic group relative to basal group, and vitamin C treatment reduced this number (Panel B). Vitamin C also reduced the number of macrophages as well as lymphocytes in the BALF (Panels C and D) when compared to fibrotic group.

The phenotypic analysis of leukocytes by flow cytometry assay showed increased percentage of granulocytes in fibrotic group relative to basal group, and vitamin C treatment reduced this percentage (Panel E). PQ administration also increased the percentage of lymphocytes in the BALF, and vitamin C treatment reduced these cells (Panel F). In Panel G we can observe that PQ administration reduced the percentage of alveolar dendritic cells into the BALF when compared to basal group and vitamin C did not modify this reduction.

### Vitamin C treatment reduced levels of IL-6, IL-17 and TGF-β in the lung homogenates of PQ-induced fibrosis

**Fig 2:**
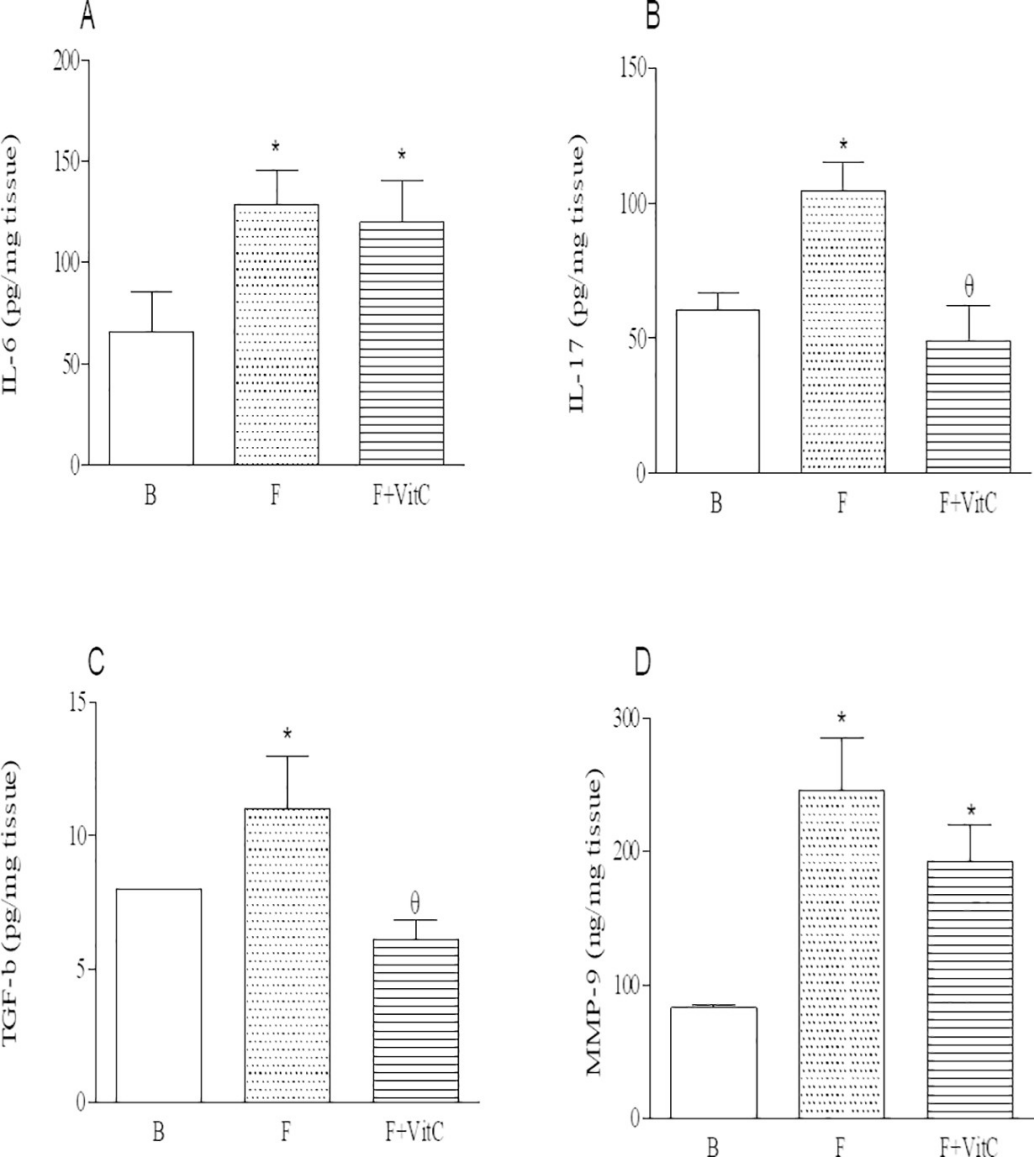
Vitamin C treatment reduces the levels of IL-6, IL-17 and TGF-β in the lung homogenates of PQ-induced fibrosis. Groups of mice were submitted to PQ or vehicle injection and after 7 days the animals were treated with vitamin C or vehicle daily until day 14th. Twenty-four hours after the last treatment the level of cytokines was quantified in the lung homogenates. Data represent the mean ± SEM of 6 animals. *P<0.05 in relation to B group; ^θ^P <0.05 in relation to F group.

Inflammatory and pro-fibrotic mediators secreted by local and migrated cells perform crucial role in the advance of fibrosis disease. Lung homogenates from fibrotic mice presented increased levels of IL-6, IL-17, TGF-β and MMP-9 relative to B group. Vitamin C treatment reduced the levels of IL-6, IL-17 and TGF-β, but not MMP-9 relative to non-treated mice (Fig 2, Panels A-D).

### Vitamin C treatment decreased collagen deposition in the lung of fibrotic mice

**Fig 3:**
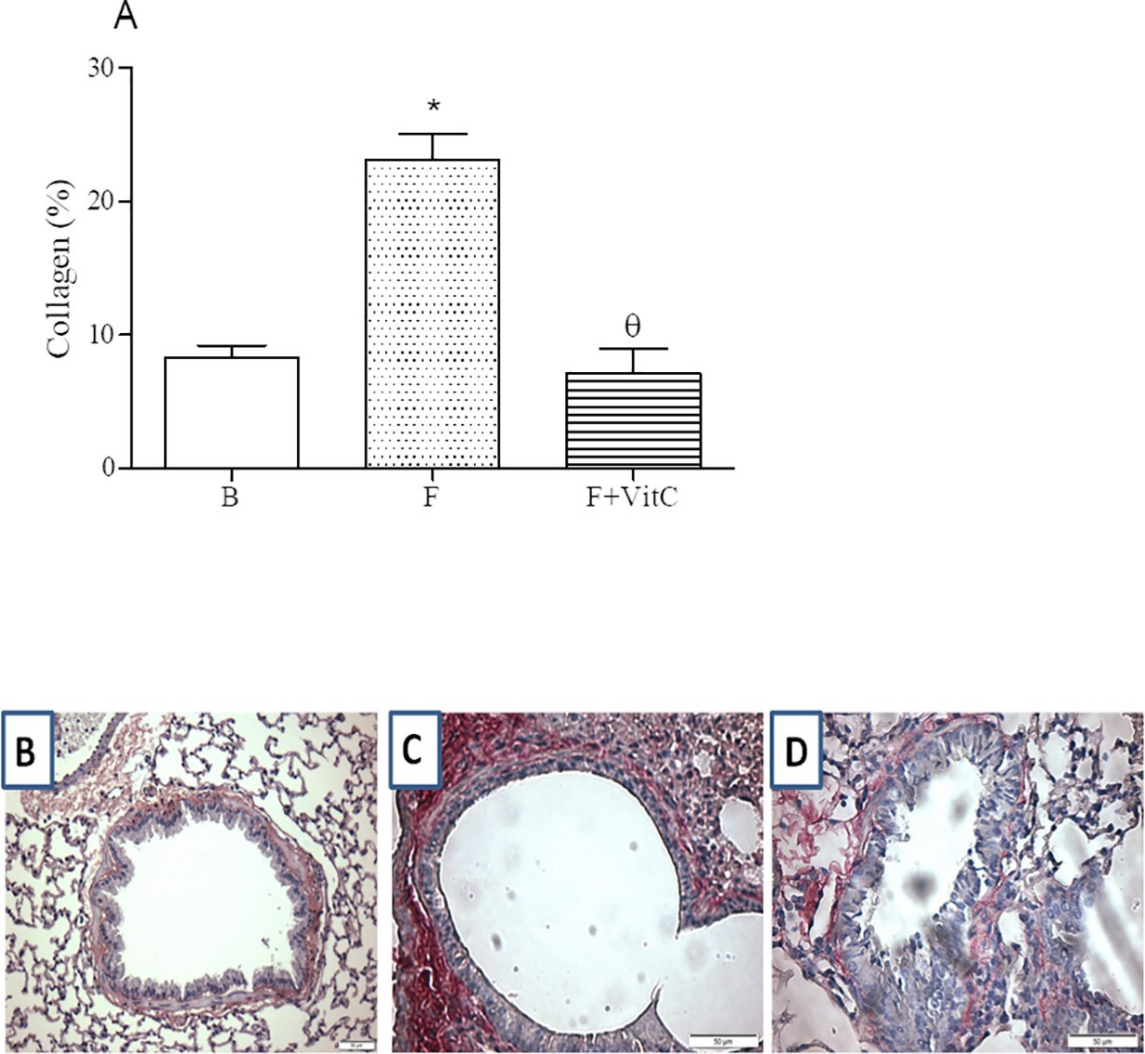
Vitamin C treatment reduces collagen deposition in the lung of PQ- induced fibrosis. Groups of mice were submitted to PQ or vehicle injection and after days the animals were treated with vitamin C or vehicle daily until day 14th. Twenty-four hours after the last treatment the collagen deposition was determined in the lung tissue. Data represent the mean ± SEM of 6 animals. *P<0.05 in relation to B group^; θ^P<0.05 in relation to F group.

Our data, as expected, showed the accumulation of collagen increment in the lung tissue of fibrotic mice relative to basal group (Fig 3, Panels A-D), and we also showed that vitamin C treatment reduced significantly the collagen deposition in the lung.

### Vitamin C treatment increased the activity of antioxidant enzymes in the lung homogenates of fibrotic mice

**Fig 4:**
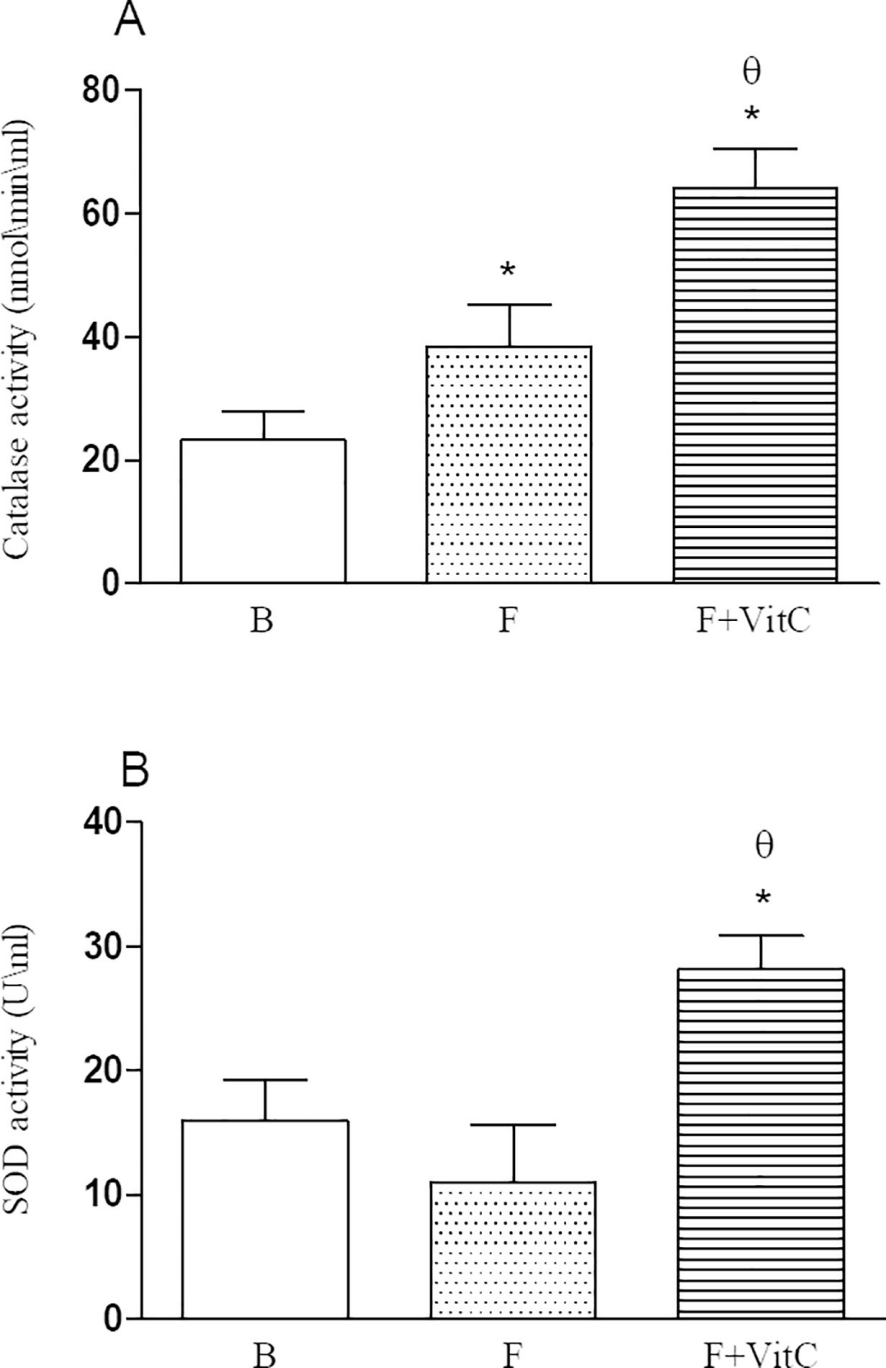
Vitamin C treatment increases antioxidant enzymes in the lung tissue of PQ-induced fibrosis. Groups of mice were submitted to PQ or vehicle injection and after 7 days the animals were treated with vitamin C or vehicle daily until day 14th.Twenty-four hours after the last treatment the collagen deposition was determined in the lung tissue. Data represent the mean ± SEM of 6 animals. *P<0.05 in relation to B group; ^θ^P <0.05 in relation to F group.

Considering that oxygen reactive species are important mediators secreted during fibrosis disease, we decide to investigate if vitamin C treatment improves the antioxidant mechanism. Lung homogenates from fibrotic mice treated with vitamin C presented an increment in the activities of superoxide dismutase and catalase in relation to non-treated mice (Fig 4, Panels A and B).

### Vitamin C treatment did not alter the maximum contractile response to methacholine in fibrotic mice

**Fig 5:**
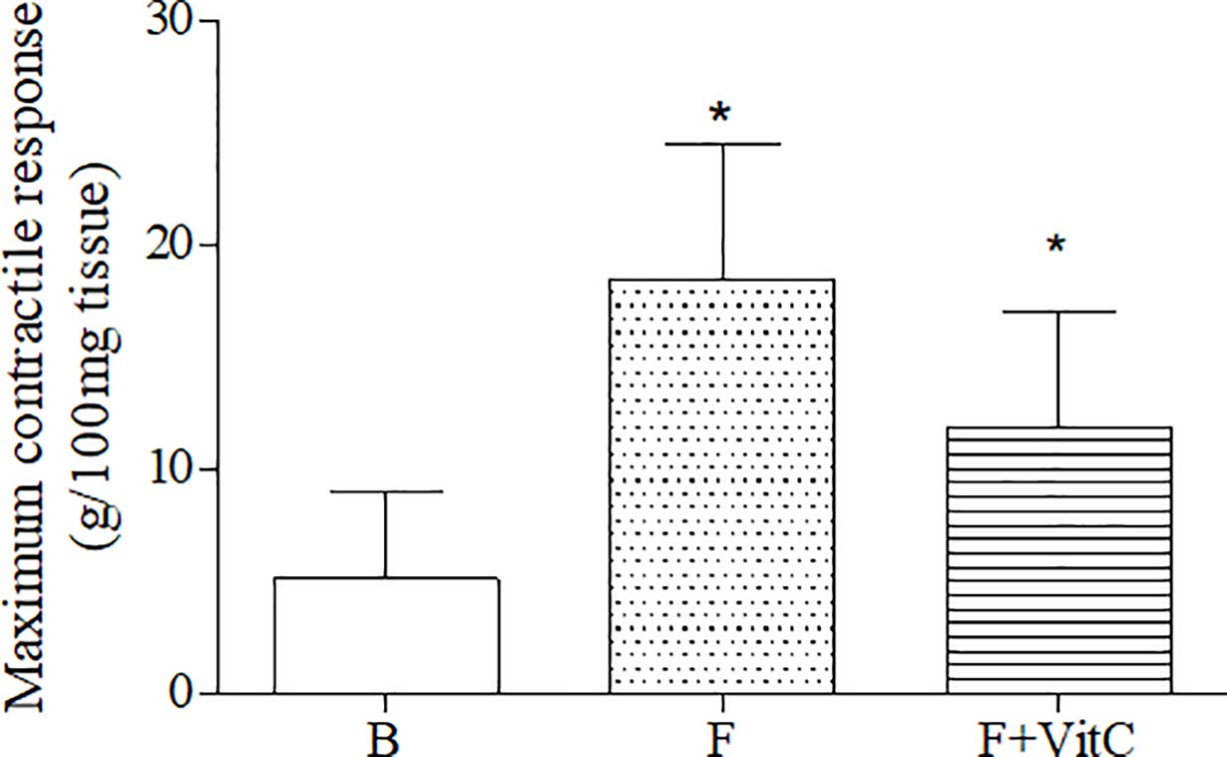
Vitamin C treatment did not alter the maximum contractile response to methacholine. Groups of mice were submitted to PQ or vehicle injection and after 7 days the animals were treated with vitamin C or vehicle daily until day 14th.Twenty-four hours after the last treatment the tracheal responsiveness was evaluate. Data represent the mean ± SEM of 6 animals. *P<0.05 in relation to B group.

Fig 5 showed that vitamin C did not reverse the elevated contractile response to MCh induced by paraquat. Paraquat induce increased tracheal responsiveness when compared to trachea of non-treated animals (B group).

## Discussion

PQ intoxication is a big health problem as it causes accidental or intentional environmental/occupational intoxications leading to severe toxic effects, including pulmonary fibrosis. The mechanisms of the intoxication are complex and no effective therapeutical approach is currently available [31,32]. When considering that an increased oxidative stress may be the toxic mechanism of a PQ intoxication, the researchers of this study have tested vitamin C as an antioxidant agent, in order to halt this PF condition. Indeed, the investigators have pointed out that the blockade of the oxidant pathways, one week after the PQ exposure, was a remarkable therapy for preventing the development of PF.

The data has shown that two weeks after the PQ injections, there occurred a mobilization of the neutrophils, the macrophages and the lymphocytes into the BAL, with an increased generation of IL-6, IL-17, TGF-β and MMP-9, together with a collagen deposition, as well as with an elevated smooth muscle responsiveness. Probably, such changes contributed to the reduction of the O_2_/CO_2_ exchange, although that was not evaluated at this time in this study. However, in an earlier study, the current researchers have shown that an increased lung elastance was induced by bleomycin and that this was attributed to an elevated collagen deposition [33].

Neutrophils are short-lived cells in the circulation that are continuously produced by the bone marrow and delivered to the blood. Nevertheless, this process is exacerbated during an innate inflammation and the blood neutrophils rapidly migrate into an inflamed tissue to kill the inductor agent of the injury. Conversely, the lymphocytes are long-lived cells that accord to distinct phenotypes and they reach the tissues during an acquired inflammation. Hence, it is possible to infer that a PQ-induced PF activates these complex cellular mechanisms, as the recruitment of these cells involves distinct intracellular pathways and a chemical mediation. The vitamin C treatments in the mice halted the leukocyte recruitment, which could suggest a direct action of vitamin C in these cells. This data has corroborated with that obtained by Novac et al., [34], where vitamin C injections impaired the neutrophil migration into the pleural cavities of the mice after a zymosan activation. Nevertheless, strong data in the literature shows that the neutrophils accumulate a great amount of vitamin C and that this is useful for protecting the intracellular mechanisms from a high oxidative stress during an inflammation. In this context, vitamin C favors chemotaxis and phagocytosis and it enhances a microbial killing, while at the same time, it also facilitates apoptosis and a clearance [25,35,36,37,38,39,40,41,42,43,44]. Similarly, the lymphocytes also accumulate vitamin C, modulating the cell proliferations, as well as the T and B cell functions [25]. Therefore, the reduced influx of cells into the alveolar space after a vitamin C treatment may be due to the lower levels of the pro-inflammatory cytokines that are secreted by the resident or the migrated cells. Indeed, the studies that were conducted by Zhao et al. [9] have indicated that mitochondrial fission is involved in a PQ-induced apoptosis and that vitamin C treatments *in vitro* attenuated this PQ-induced apoptosis. Thus, this current study might hypothesize that vitamin C reduced the number of apoptotic cells into the lung, abrogating the course of lung fibrosis. This hypothesis will be investigated in the future.

The role of vitamin C on the production of the inflammatory mediators is controversial and the effects seem to be dependent on the cell type and/or the inflammatory conditions [25]. Indeed, it has been reported that high doses of vitamin C that were used to treat a paraquat intoxication can promote an aggravated production of hydroxyl radical (OH(•)), by interacting with a preexisting PQ(+•)/H_2_O_2_ system, exacerbating the apoptotic killing of the cells [45].

This present study observed lower levels of IL-17 and TGF-β in the lung homogenates of the fibrotic mice that were treated with vitamin C. The IL-17 levels displayed pleotropic inflammatory effects, driving the migration of the inflammatory cells into the inflamed tissues [46,47]. Thus, the researchers have supposed that ascorbic acid may act mainly on these cells, impairing their migration into the lungs and/or by directly reducing the IL-17 secretions. TGF-β is a pro-fibrotic cytokine that induces the proliferation, the migration and the differentiation of fibroblasts to myofibroblasts, as well as the extracellular matrix production and the chemotactic signals for the leukocytes [48,49]. The current study’s results also showed reduced levels of TGF- β after the vitamin C treatments. These reductions can be linked to a lower collagen production, since TGF-β is a mediator of the collagen production and with the consequent rearrangement and deposition in the inflamed tissues [50].

Moreover, high levels of MMP-9 in the lungs after the PQ exposures were detected, which is also a hallmark of fibrosis. The metalloproteinases (MMPs) also played an important role in the PF conditions, by degrading the extracellular matrix–consequently, by attracting the inflammatory cells to the site of the injuries, perpetuating the cycles of inflammation and remodeling [51]. The MMP-9 levels are elevated in patients with an idiopathic lung fibrosis [52]. The schedules of vitamin C that were carried out here in this research did not reverse the high levels of MMP-9. On the other hand, Gupta et al. [53] showed that vitamin C treatments impaired the increment of MMP-9 in the lung cells that were evoked by cigarette smoking. Corroborating with this study, Liu et al. [54] showed that vitamin C reduced the levels of TNF, IL-17 and TGF- β mRNA, after a lung fibrosis that was paraquat-induced. However, the focus of that study was not to evaluate the effects of vitamin C. In their study, the authors showed the effects of Salvianolic acid B on a lung pathology, a collagen deposit, the stress marker MDA, the superoxide dismutase activity and on the cytokine release (TGF-β, IL17 and TNF), demonstrating the importance of the TGF/SMAD3 pathway, in its protective effect on a paraquat intoxication. It was important to consider the differences between this present research and the protocols of the study of Liu and coworkers, on inducing fibrosis, as well as on treating the animals with vitamin C. Liu et al. [54] evaluated the lung fibrotic injuries that were induced in the mice by a single intragastrical administration of 300 mg/kg PQ, while this study used 10 mg/kg PQ by the intraperitoneal route. Toxicologically, the dose and the route are directly linked to the effects, thus, it is important to conduct studies using lower doses of the toxicants. Similarly, it is necessary to evaluate the efficacy of the treatments, when the intoxication occurs with a high or a low dose, because the pathways triggered can be different, dependent upon the dose and the route.

It is well established that an excessive production of the reactive oxygen species (ROS) causes oxidative stress, impacting with important effects on several biological molecules, such as the lipids, the DNA and the proteins [55]. In this context, clinical studies have suggested that oxidative stress also plays an important role in PF [23]. Those patients with PF had oxidative stress, which then triggered the intracellular signaling, inducing fibroproliferation and the expression of the pro-fibrotic factors [20,21].

Oxidative stress can be modulated by the antioxidant defense mechanisms, such as by an enzymatic (superoxide dismutase, catalase and glutathione redutase) treatment, as well as by a non-enzymatic (ascorbic acid and alpha-tocopherol) treatment [55]. When considering that paraquat induces lung fibrosis by the oxidative stress-mechanisms, this study has hypothesized that an increment in the antioxidant system that is caused by vitamin C would be helpful in reducing the reactive oxygen species, thus, ameliorating the lung fibrosis response. As a result, the current researchers have evaluated the role of vitamin C on the antioxidant enzymes. The data showed elevated levels of SOD and catalase after the vitamin C treatments. Therefore, vitamin C improved the antioxidant defense mechanisms and such effects might be responsible, at least in part, for the lower inflammatory and fibrotic responses.

It is well established that during lung fibrosis, there is a reduction in the function of the lungs. However, there are not studies showing alterations directly in the reactive airways. When considering that lung fibrosis is characterized by an elevation of the inflammatory cells in the airways and that these cells can release several mediators that can modulate the smooth muscle contractions, it was evaluated whether these mediators could contribute to the alterations in the smooth muscle, hence, contributing to the reductions of air flux in the airways. In fact, elevated tracheal reactivities to methacholine after the fibrosis inductions were shown. Thus, this result was important, showing that not only the lung functions were altered during the fibrosis, but alterations in the smooth muscle also occurred and that they can play important roles in this type of a pathology. These evaluations have not been considered up until now. Vitamin C was capable of reversing many parameters, except for the tracheal contractile response. This is plausible considering its mechanisms. The contractile response is modulated by several mediators that are possibly not modified by vitamin C, including the leukotrienes and other mediators.

In conclusion, these results have shown that oxidative stress may be a mechanism of inflammation, together with a collagen deposition in the lungs, after a PQ-induced lung fibrosis. Vitamin C seems to be an important tool for treating this intoxication. It is noteworthy to mention that vitamin C is cheap and could be a feasible agent to be employed as a therapy.

## Supporting information

S1 File Data supporting. (DOCX)
