# Supplementary material for: Beneficial effects of ascorbic acid to treat lung fibrosis induced by paraquat
Source: PLoS One. 2018 Nov 5;13(11):e0205535. doi: 10.1371/journal.pone.0205535 (PMC6218022; doi:10.1371/journal.pone.0205535)
Supplement: S1 File — (DOCX) [file pone.0205535.s001.docx]

Data supporting

Number of total cells in the BAL

| B | F | F+VitC |
| --- | --- | --- |
| 6,0 | 20,00 | 4,50 |
| 5,0 | 12,50 | 6,00 |
| 4,0 | 17,75 | 4,00 |
| 6,0 | 17,50 | 6,75 |
| 5,0 | 13,75 | 4,67 |
| 4,9 | 11,25 | 5,79 |
|  | 12,50 |  |

Number of neutrophils in the BAL

| B | F | F+ VitC |
| --- | --- | --- |
| 0,50 | 8,00 | 2,67 |
| 0,60 | 9,76 | 3,00 |
| 0,45 | 8,40 | 3,30 |
| 0,50 | 7,99 | 3,00 |
| 0,45 | 8,10 | 3,10 |
| 0,55 | 9,00 | 2,87 |

Number of macrophages in the BAL

| B | F | F + VitC |
| --- | --- | --- |
| 3,50 | 4,80 | 2,00 |
| 3,00 | 5,00 | 2,30 |
| 2,90 | 4,60 | 2,50 |
| 3,20 | 5,10 | 1,67 |
| 3,00 | 4,97 | 1,90 |
| 3,17 | 5,18 | 1,88 |

Number of lymphocytes in the BAL

| B | F | F + VitC |
| --- | --- | --- |
| 1,00 | 1,80 | 1,00 |
| 0,99 | 1,90 | 0,98 |
| 1,20 | 2,00 | 0,90 |
| 1,10 | 2,10 | 1,10 |
| 1,34 | 1,77 | 1,09 |
| 0,87 | 2,04 | 0,86 |

Lyg6+

| B | F | F+ VitC |
| --- | --- | --- |
| 10,24 | 13,10 | 10,73 |
| 5,66 | 11,29 | 8,91 |
| 10,04 | 10,76 | 5,36 |
| 9,62 | 10,41 | 9,05 |
| 9,54 | 11,00 | 7,87 |
| 6,00 | 10,67 | 8,10 |

Cd11b+

| B | F | F + VitC |
| --- | --- | --- |
| 2,45 | 1,57 | 1,75 |
| 1,28 | 1,31 | 1,77 |
| 3,83 | 1,91 | 1,15 |
| 3,92 | 1,12 | 3,46 |
| 2,83 | 1,25 | 2,30 |
| 3,00 | 1,33 | 1,00 |

Cd3+

| B | F | F + VitC |
| --- | --- | --- |
| 6,00 | 16,40 | 9,83 |
| 6,42 | 9,93 | 8,98 |
| 10,03 | 10,99 | 6,48 |
| 8,26 | 10,39 | 9,22 |
| 8,05 | 16,33 | 7,65 |
| 10,12 | 11,00 | 9,00 |
| IL-6   \| B \| F \| F+VitC \| \| --- \| --- \| --- \| \| 85,333340 \| 111,702100 \| 140,000000 \| \| 46,615380 \| 132,051300 \| 179,655200 \| \| 83,337470 \| 160,500000 \| 55,510200 \| \| 45,262520 \| 83,000000 \| 103,222200 \| \| 86,665350 \| 92,435900 \| 121,126800 \| \| 42,998730 \| 192,381000 \| 100,234900 \| |  |  |

IL-17

| B | F | F+VitC |
| --- | --- | --- |
| 66,600000 | 131,025600 | 17,567570 |
| 54,153850 | 123,666700 | 91,818180 |
| 55,229990 | 81,125000 | 15,063290 |
| 53,234710 | 79,487180 | 28,965520 |
| 64,987680 | 107,857100 | 25,306120 |
| 62,786200 |  | 88,111110 |
|  |  | 76,478870 |

MMP-9

| B | F | F+VitC |
| --- | --- | --- |
| 87, | 223, | 241, |
| 80, | 380, | 261, |
| 82, | 178, | 281, |
| 78, | 171, | 117, |
| 81, | 280, | 130, |
| 80, | 219, | 207, |
|  |  | 113, |

TGF-beta

| B | F | F+VitC |
| --- | --- | --- |
| 8,00 | 19,00 | 5,6 |
| 8,00 | 8,43 | 5,3 |
| 8,00 | 9,00 | 3,4 |
| 7,40 | 9,00 | 7,5 |
| 8,21 | 9,10 | 6,9 |
| 7,80 | 9,33 | 8,1 |

Collagen

| B | F | F+VitC |
| --- | --- | --- |
| 10,00 | 26,36 | 5,13 |
| 8,00 | 21,09 | 14,00 |
| 7,00 | 18,52 | 3,68 |
| 9,00 | 26,45 | 7,64 |
| 7,98 | 25,44 | 5,29 |
| 10,02 | 20,00 | 5,66 |

Catalase

| B | F | F+VitC |
| --- | --- | --- |
| 38, | 19, | 56, |
| 13, | 21, | 74, |
| 20, | 39, | 40, |
| 30, | 41, | 69, |
| 15, | 49, | 85, |
|  | 62, | 61, |

SOD

| B | F | F+VitC |
| --- | --- | --- |
| 23, | 8,00 | 15,00 |
| 10, | 2,60 | 23,00 |
| 11, | 27,80 | 37,00 |
| 20, | 2,14 | 29,00 |
| 12, | 22,89 | 32,00 |
| 22, | 2,43 | 29,00 |
| 10, |  | 32,00 |

Tracheal reactivity

| B | F | F+VitC |
| --- | --- | --- |
| 1,25 | 24,5 | 6,80 |
| 9,00 | 12,5 | 17,00 |
| 3,00 | 35,0 | 5,00 |
| 12,00 | 20,0 | 15,00 |
| 1,30 | 5,0 | 24,00 |
| 7,00 |  | 2,00 |
